# The impact of plasma-rich platelet injection to perianal sphincters on incontinence and quality of life in patients with rectal cancer after low anterior or intersphincteric resection: a prospective cohort study

**DOI:** 10.1007/s10151-024-02989-1

**Published:** 2024-08-14

**Authors:** M. Haksal, M. S. Akın, E. Karagoz, M. Kocak, E. Korkut, R. Shahhosseini, I. Gögenur, M. Oncel

**Affiliations:** 1 Department of General Surgery, Medipol Mega Hospital, Istanbul Medipol University Medical School, TEM Avruoa Otoyolu Göztepe Çıkışı, No: 1 Bağcılar, 34214 Istanbul, Turkey; 2 Department of Gastroenterology, Istanbul Medipol University Medical School, Istanbul, Turkey; 3 Department of Biostatistics and Medical Informatics, Istanbul Medipol University Medical School, Istanbul, Turkey; 4 Faculty of Medicine, Istanbul Medipol University Medical School, Istanbul, Turkey; 5 Department of Clinical Medicine, Center for Surgical Science, Zealand University Hospital, Copenhagen University, 2200 Copenhagen N, Denmark

**Keywords:** Plasma rich platelet, Incontinence, Rectal cancer, Low anterior resection, Intersphincteric resection

## Abstract

**Background:**

Incontinence is not rare after rectal cancer surgery. Platelet-rich plasma may promote tissue repair and generation but has never been tested for the treatment of anal incontinence. This study evaluated the impact of platelet-rich plasma injection on the severity of incontinence and quality of life after low rectal cancer surgery.

**Methods:**

This is a prospective cohort proof of concept study in a colorectal cancer institution. Patients had undergone low anterior or intersphincteric resection for low rectal cancer and had a Wexner score > 4. Ten milliliters of platelet-rich plasma were injected into the internal and external sphincters under endoanal ultrasound (EAUS) guidance. Primary outcome measure was > 2 point improvement in Wexner score (improved group). The patients were assessed with endo-anal ultrasound examination, manometry, the Wexner Questionnaire and SF-36 Health Surveys, and patients were asked whether they used pads and antidiarrheal medications before and 6 months after PRP injection.

**Results:**

Of 20 patients included in the study, 14 (70%) were men, and the average age was 56.8 (SD = 9.5) years. No statistically significant difference was found in Wexner scores before and after PRP injection (*p* = 0.66). Seven (35%) patients experienced a > 2 point improvement in Wexner score. Rectal manometry demonstrated improved squeezing pressure (*p* = 0.0096). Furthermore, physical functioning scoring (*p* = 0.023), role limitation (*p* = 0.016), emotional well-being (*p* = 0.0057) and social functioning (*p* = 0.043) domains on the SF-36 questionnaire improved. One (5%) and three (15%) patients stopped using pads and antidiarrheal medications.

**Conclusion:**

Platelet-rich plasma injection does not restore Wexner scores, but more than one-third of patients may benefit from this application with an improvement of > 2 points in their scores. Platelet-rich plasma injection may improve squeezing pressure and certain life quality measures for incontinent patients after rectal cancer surgery.

**Supplementary Information:**

The online version contains supplementary material available at 10.1007/s10151-024-02989-1.

## Introduction

Surgery for rectal cancer can be challenging, especially when the tumor is located at the lower rectum and patients desire sphincter preservation to avoid a stoma. Unfortunately, sphincter preservation can result in symptoms like fecal incontinence, as perfect continence is only achieved in about half of these cases [1–3]. While the exact causes of incontinence are not yet fully comprehended, it may arise from a variety of causes including direct and indirect damage to the anal sphincter [2, 4]. Fortunately, various techniques exist to improve quality of life (QoL) for these patients, such as different types of reservoirs, sacral or tibial nerve stimulation, pelvic floor rehabilitation with biofeedback and transanal irrigation. While these approaches can alleviate certain symptoms, they are not universally applicable because of factors such as the transient effect of the treatments and poor patient tolerance [5–7].

Platelet-rich plasma (PRP) is a biological product obtained from the plasma fraction of autologous blood [8]. With a higher concentration of platelets and clotting factors, PRP contains several growth factors, cytokines, chemokine and cell-adhesion molecules that activate and stimulate the synthesis of essential products involved in the healing process and tissue proliferation and regeneration [8–11]. PRP has been used in medicine, especially in orthopedics and ophthalmology for various purposes, including immunomodulation, regeneration induction and pain relief, and has an adjunct role in a variety of regenerative medical treatments, including perianal fistula and stress urinary incontinence [12–14]. As such, we conducted this prospective proof of concept trial to examine the effectiveness of PRP injection in treating anal incontinence after low anterior resection (LAR) or intersphincteric resection (ISR).

## Materials and methods

This is a single-center prospective cohort study approved by the Istanbul Medipol University Ethics Committee (66,291,034–29-11.05.2015) in compliance with the Declaration of Helsinki. Written consent was obtained from all patients, and patient enrollment and follow-up were completed between 2018 and 2021.

### Patients and inclusion and exclusion criteria

A thorough review of charts and the departmental database was conducted to identify patients complaining of incontinence among those who had undergone a LAR or ISR for rectal cancer at our institution and had had incontinence complaints during their last office visit. To participate in the study, patients had to meet certain criteria, including being > 18 years of age, providing written consent, having an adequate time interval after the initial operation (> 12 months), showing no evidence of local recurrence or systemic metastasis, having a Wexner score > 4 and adhering to conservative treatments including diet regulation, fiber support, loperamide, adequate water intake (for at least 3 months) and receiving bio-feedback enforcement treatment.

To ensure that only those patients with incontinence most likely to occur because of inadequate sphincter volume or function were included in the study, patients who met any of the following criteria obtained with a manometer (Solarblue, MMS, The Netherlands) or endoanal ultrasound examination (EAUS) (BK3500, BK Medical, MA, USA) were excluded from the study.

1. Normal resting and squeeze pressures in preprocedural manometric examination.
2. Deficient external sphincter volume, defined as external sphincter < 16.1 mm long, 6.8 mm thick or 6.3 cc in volume in endoanal ultrasound examination [15–17], since it is technically difficult to accurately inject the material in such muscles, or any level full-thickness external muscle tear due to several reasons diagnosed by EUS examination.
3. An increase in first sensation feeling (> 23 cc), which showed loss of rectal compliance [18, 19].
4. A decrease in maximum tolerable volume (< 60 cc), which revealed insufficient neo-rectum volume [20].

Other exclusion criteria included the following: pregnancy or breast-feeding, presence or suspicion for local recurrence or metastasis at any location, undergoing chemotherapy, patient refusal, pre-existing incontinence before the initial operation, contraindications for PRP application such as active infection, thrombocytopenia, severe anemia (hemoglobin level < 7 g/dl) or history of allergy to bovine thrombin.

### Measures

Patient-Reported Outcomes: The study used the Wexner Incontinence Questionnaire and SF-36 Health Survey to assess patient-reported outcomes. Both instruments were validated for the Turkish population [21, 22]. These questionnaires were administrated both before and 6 months after the PRP application. Use of pads and consumption antidiarrheal medications before and after the intervention were noted. Wexner scores and pad use were evaluated at least 48 months after PRP application to analyze long-term results.

#### Objective measures

One of the investigators (MCH) measured the length, thickness and volume of the internal and external muscles using endoanal EAUS (Flex Focus 800, BK Medical, Burlington, MA, USA) before PRP application. An experienced gastroenterologist (EK) performed manometric examinations (Solar Blue Anorectal Manometry System [Air Charged Catheter], MMS, Medical Measurements Systems, The Netherlands) to determine anal resting pressure, maximum squeezing pressure, isolated squeezing pressure, first sensation volume, sensation of defecation pressure and maximum tolerable volume. The investigations were repeated 6 months after PRP application. To standardize the evaluations throughout the study period, the same investigators performed EAUS and manometry examinations.

The study database included demographics, weight, height, and body mass index, comorbidities such as hypertension and diabetes, information about index tumor and treatment, and the period between the initial operation (or stoma closure if there was a diverting stoma) and PRP injection.

### PRP preparation and procedure

The recommendations of the manufacturer (Minos PRP 20 cc, NeoGenesis Co., South Korea) were followed during PRP preparation. A total of 18 cc blood was drawn into injectors containing 2 ml sodium citrate. The inverted blood was placed in the PRP kit and centrifuged at 1480 rpm for 4 min. The process left 10 ml of yellow liquid enriched with platelets above the blend, and this plasma was extracted from the kit using a special syringe.

PRP application was completed under general anesthesia. Under EAUS guidance, 10 ml PRP was injected into four quadrants of the internal and external sphincter using an 18-gauge injector. The primary targets for injection were both the internal and external muscles. If the internal muscle bundles could not be well recognized and cannulated accurately, the injection was applied to the external muscles. While injecting, the needle was moved back and forward and up and down to spread the liquid within the muscle fibers. Patients were discharged from the hospital the same day within 4 to 8 h after the procedure.

### Analyzed measures

The study scrutinized the changes in patient-reported outcomes and objective measures from baseline to 6 months after PRP injection. A threshold of > 2 point progress in Wexner score has been accepted as significant improvement as this was previously shown to reflect a clinical impact [23, 24]. These patients were included in the ‘improved group,’ the others in the ‘failed group.’ The number of patients who discontinued use of pads or antidiarrheal medications was documented. Patient- and disease-related factors that could anticipate a > 2-point progress in Wexner score were investigated.

### Statistics

Categorical variables were expressed as frequencies and percentages and continuous variables as mean and standard deviations or median and interquartile range. To investigate the association of two categorical factors, chi-square or Fisher exact test was used as appropriate. To compare the distribution of continuous variables among the categorical variable levels, we used Wilcoxon-Mann-Whitney or Kruskal-Wallis test as appropriate. To assess the significance of change between the two time points, namely baseline and 6-month visits, Wilcoxon signed rank test was used. A *p* value < 0.05 was accepted as significant. No multiple testing adjustment was made on the *p*-values; therefore, the results must be considered within a hypothesis-generating context.

## Results

### Patient characteristics

A total of 23 patients were screened, and 3 were excluded since they did not fulfill the EAUS or manometer inclusion criteria (increased first sensation feeling [*n* = 2] or normal squeeze pressure [*n* = 1]). So, the study included 20 patients (14 [70%] men) with a mean (± SD) age of 56.8 ± 9.5 years and a mean (± SD) of body mass index of 29.6 ± 3.4 kg/m^2^. Ten (50%) patients had comorbidities including hypertension (*n* = 8), diabetes mellitus (*n* = 3) and others (*n* = 3). Among the patients, 19 (95%) received neoadjuvant chemoradiation (45–54 Gy for 25 days, *n* = 12) or radiation (25 Gy for 5 days, *n* = 7) therapy. All the patients took postoperative chemotherapy for a mean (± SD) of 8 ± 4.1 cycles for 5.3 ± 2.3 months. Laparoscopy was preferred in 19 (95%) cases, but 1 (5%) required conversion. One case (5%) was operated on using an open technique. The transanal total mesorectal excision technique was not preferred for any of the study patients. The cancer was located < 2 cm from the dentate line in 16 (80%) cases and 2–5 cm in 4 (20%). Intersphincteric resection with handsewn coloanal anastomosis or low anterior resection with stapled anastomoses was performed in 16 (80%) and 4 (20%) patients, respectively. A straight anastomosis was preferred for all cases. Eighteen (90%) patients had a diverting ileostomy at the time of initial surgery, which was reverted in 3 to 6 months.

During the postoperative period, complications were observed in six (30%) cases, including atelectasis and fever (*n* = 3, 15%), wound infection (*n* = 2, 10%), urinary infection (*n* = 1, 5%) and pneumonia (*n* = 1, 5%). All were measured as Clavien-Dindo ≤ 3, and complications were reported related to the anastomosis. In the long term, mucosal prolapse of neorectum occurred in two patients (10%) and mild anal stenosis which necessitated anal dilatation in one (5%). Pathological examinations revealed ypT0, ypT1, ypT2 and ypT3 tumors in 3 (15%), 1 (5%), 10 (50%) and 6 (30%) cases, and 19 (95%) were node negative. The median (Q1–Q3) interval between the initial operation and PRP application was 32.5 (21.5–72.5) months. The patients were asked to continue regular pelvic exercises, and a single patient received biofeedback treatment before PRP injection. No patients received transanal irrigation treatment and/or sacral/pretibial nerve stimulation therapy before or after PRP application.

At baseline, the mean (± SD) baseline Wexner score was 17.5 ± 3.3 and > 7 in all patients; relatedly, all were using pads and on antidiarrheal medications.

### PRP injection outcomes

All patients were discharged on the same day, and no complications related to PRP application were observed.

### Patient-reported outcomes

**Fig. 1:**
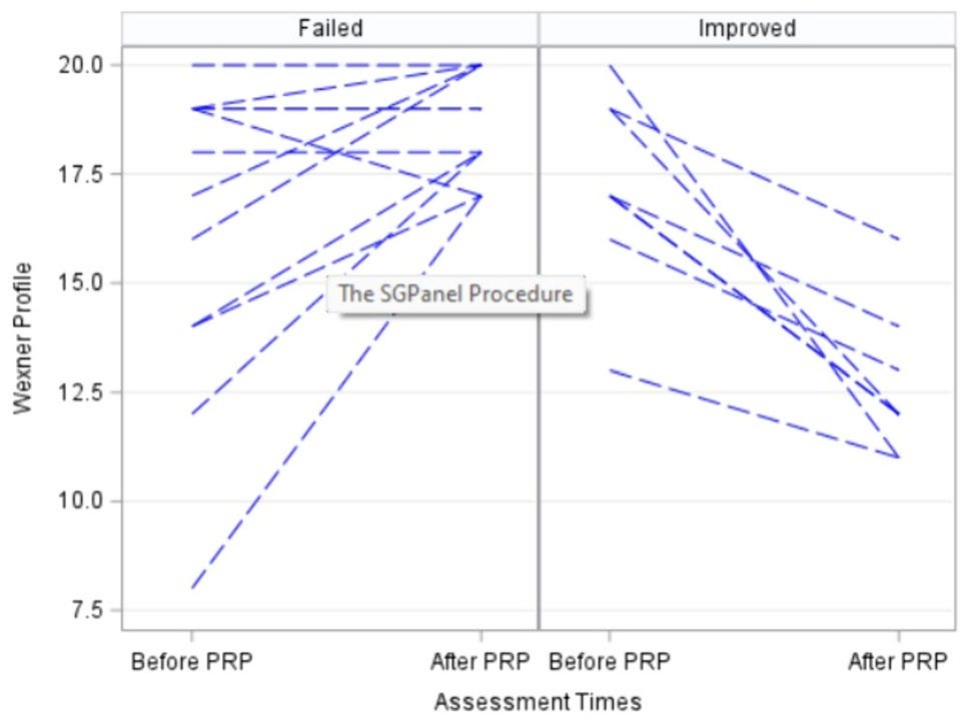
Changes in Wexner scores in 20 cases; failure group (*n* = 13) on the left and success group (*n* = 7) on the right

**Fig. 2:**
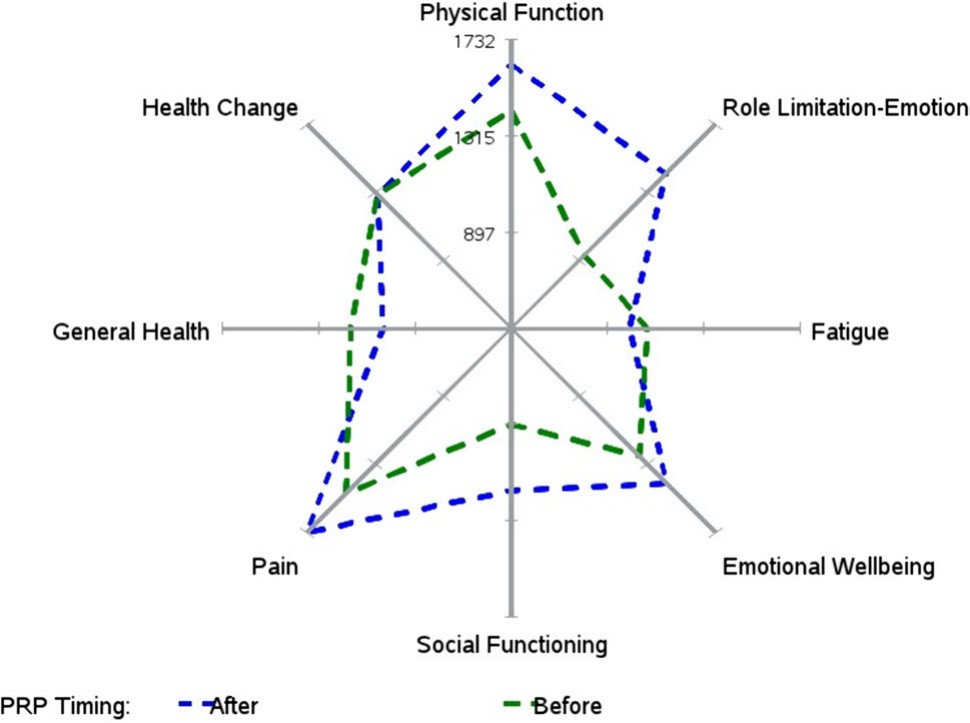
Changes in SF-36 measures, statistically significant improvements in physical functioning, role limitation due to emotional problems, emotional well-being and social functioning measures

No statistically significant difference was found in the Wexner scores between PRP injections (*p* = 0.66) either before or after treatment. Seven (35%) patients demonstrated a ≥ 2 point improvement in their initial Wexner scores after PRP injection (improved group). Among the failed group (*n* = 13, 65%), the Wexner scores worsened in six (46.2%), were unchanged in three (23.1%) and increased ≤ 2 points in four (30.8%) patients (Fig. 1). SF-36 QoL questionnaires (*n* = 20) revealed statistically significant enhancements in physical functioning (*p* = 0.023), role limitations (*p* = 0.016), emotional well-being (*p* = 0.0057) and social functioning (*p* = 0.043) scores (Table 1, Fig. 2). Among the improved patient group, one (5%) had stopped using pads and three (15%) had stopped taking antidiarrheal medications.

**Table 1:** Comparison of EAUS and manometer measures and SF-36 scores before and 6 months after PRP injection

Measure	Before PRP	After PRP	Difference	*p*
Incontinence level				
Wexner score	17.5 (15–19)	17 (12.5–19)	0 (– 3 to 3)	0.66
Patient-reported outcomes				
SF-36 Questionnaire				
Physical functioning	75 (52.5–92.5)	92.5 (65–100)	10 (0–17.5)	0.023
Role limitation due to physical health	37.5 (0–50)	75 (12.5–75)	0 (– 62.5–25)	0.14
Role limitation due to emotional problems	33.3 (33.3–66.7)	83.4 (49.7–100)	16.7 (0–66.7)	0.016
Energy fatigue	47.5 (37.5–75)	50 (40–65)	0 (– 10 to 7.5)	0.62
Emotional well-being	64 (54–70)	74 (66–78)	8 (0–16)	0.0057
Social functioning	37.5 (25–68.8)	62.5 (43.8–75)	12.5 (0–37.5)	0.043
Pain	77.5 (55–95)	100 (77.5–100)	12.5 (0–22.5)	0.061
General health	57.5 (50–70)	55 (40–62.5)	0 (– 20–5)	0.1
Health change	75 (50–75)	75 (50–75)	0 (– 25 to 25)	1.00
Objective measures				
EAUS				
External sphincter				
Length	38.1 (34.8–44.8)	40.5 (36.4–45.3)	1.2 (– 0.7 to 2.7)	0.12
Thickness	7.4 (7–8.4)	7.8 (7–8.5)	-0.1 (– 0.3 to 0.9)	0.64
Volume	6.8 (6.6–7.1)	7 (6.5–7.3)	0.2 (– 0.1 to 0.5)	0.074
Internal sphincter				
Length	38 (34.3–45.2)	37.9 (34.4–40.8)	2 (– 3.4 to 4.5)	0.6
Thickness	1.4 (1.1–2.2)	1.9 (1.2–2.2)	0 (– 0.1 to 0.1)	0.94
Manometer				
Resting pressure	31.5 (18.5–49)	31.5 (20–50.5)	1 (– 8.5 to 12)	0.65
Squeezing pressure	56 (30.5–76)	74 (58.5–111)	38.5 (5.5–56)	0.0096
First sensation	15 (10–20)	20 (15.5–32)	10 (0–16)	0.024
Maximum tolerable volume	110 (70–155)	240 (100–275)	10 (– 28.5 to 170)	0.16

Data are presented as median and (first and third quartiles)

Wexner scores were re-examined at least 4 years after PRP application. In the improved group, four out of five patients who responded to the questionnaire reported stable Wexner scores (*n* = 3) or further improvement (*n* = 1) (Table 3).

### Objective measures

Three patients declined to undergo their second EAUS and manometric examinations. Two of these patients were receiving chemotherapy for recently diagnosed metastatic disease, while the third had a cardiac deficiency. Additionally, one patient missed the manometric examination after completing his second EAUS because of travel concerns regarding his urban location. Due to limited information, manometric and EUS examinations were completed in 16 and 17 patients, respectively. Notably, manometric examinations revealed a statistically significant increase in squeezing pressure (*p* = 0.0096) (Table 1 and Fig. 2).

Type 2 diabetes was the sole predictive factor for statistically significant improvement (all 3 diabetic cases expressed significant improvement, *p* = 0.01). Additionally, although not statistically significant, female patients and those with longer internal sphincters tended to benefit more from PRP injection (*p* = 0.052 and *p* = 0.057, respectively) (Table 2).

**Table 2:** Comparisons of improved and failed groups

Analyzed factors	Improved group (*n* = 7)	Failed group (*n* = 13)	*p*
Patient-reported measures			
Gender (male/female)	3 (21.4)/4 (66.7)	11 (78.6)/2 (33.3)	0.052
Age	57.7 ± 8.2	55.2 ± 10.7	0.64
Height/weight	164.3 ± 10.3/83.1 ± 7	170.2 ± 8/83.7 ± 13.6	0.25/0.94
Body mass index	30.9 ± 1.7	28.9 ± 3.9	0.22
Concomitant disease (present/ absent)	4 (40)/3 (30)	6 (60)/7 (70)	0.28
Hypertension (present/absent)	4 (57.1)/3 (23.1)	4 (30.8)/9 (69.2)	0.59
Diabetes (present/absent)	3 (100)/4 (23.5)	0/13 (76.5)	0.01
Disease- and treatment-related measures			
T stage (0–2/3)	6 (42.9)/1 (16.7)	8(57.1)/5(83.3)	0.5
N stage (0/1,2)	7 (36.8)/0	12 (63.2)/1 (100)	0.96
Neoadjuvant (radiation/chemoradiation/none)	3 (42.9)/4 (33.3)/0	4 (57.1)/8 (66.7)/1 (100)	0.96
Operation technique (laparoscopy/open/conversion)	6 (33.3)/0/1 (100)	12 (66.7)/1 (100)/1	0.3
Resection (intersphincteric/low anterior)	6 (37.5)/1 (25)	10 (62.5)/3 (75)	0.64
Stoma (present/absent)	7 (38.9)/0	11 (61.1)/2 (100)	0.27
Interval between the initial operation and PRP injection (in months)	41.9 ± 18.7	53.4 ± 46.6	0.75
Pre-procedural measures			
External sphincter length	40.5 ± 5.2	37.6 ± 7.8	0.48
External sphincter thickness	7.7 ± 0.8	7.9 ± 1.5	0.72
External sphincter volume	7.2 ± 0.5	6.9 ± 0.5	0.26
Internal sphincter length	39.3(38–49.7)	34.4 (24.1–39.7)	0.057
Internal sphincter thickness	1.3 (1–1.4)	2 (1.2–2.2)	0.44
Initial resting pressure	32 (21–48)	31 (18–50)	0.67
Initial squeezing pressure	57 (36–60)	28 (20–84)	0.97
Initial first sensation	20 (10–20)	15 (10–20)	0.51
Initial maximal toleration volume	100 (60–80)	110 (100–140)	0.75
Initial Wexner score	18 ± 2.6	17.5 ± 3.6	0.32

Data are presented as numbers and percentages in parentheses for categorical variables and means and standard deviations or medians and first and third interquartile ranges (if standard deviation was greater than mean) for continuous variables

## Discussion

Through a proof-of-concept trial, we discovered that PRP may offer a potential solution for a specific group of patients experiencing anal incontinence following LAR or ISR. PRP derivatives have already been utilized in clinical settings to address a variety of regenerative needs including urinary incontinence, ocular ulcers, bone diseases, tendon injuries and tissue recovery after surgery [25, 26]. Several studies have underlined PRP as a safe and promising treatment for musculoskeletal maladies since it may contribute to the regeneration of the muscle cells, although its efficacy varies depending on specific indications [27]. A recent meta-analysis of prospective randomized trials has concluded that PRP is safe and effective in treating anal fistula [12]. Due to its regenerative properties, PRP can potentially be a logical treatment option for anal incontinence after rectal cancer surgery although it has yet to be tested in this context. As such, the current study hypothesized that PRP injection could be a useful option for addressing treatment of anal incontinence after LAR of ISR and enhance quality of life.

To homogenize the information and focus on fecal incontinence occurring after low anterior or intersphincteric resection, we used PRP for a highly selective group of patients. Cases with normal manometric findings were not included in the study. Patients with proven loss of rectal compliance or reduction in maximum tolerable volume or with an anatomical defect in the external muscle were also excluded since incontinence was probably not related to the functional deficiency in sphincter functions. Similarly, patients with lower external sphincter volume were not included because of the technical difficulty in accurate injection of the material in the muscles (see Table 3).

**Table 3:** Wexner scores before, 6 months and ≥ 4 years after the PRP application

Patient no.	Before application	6 months after application	≥ 3 Years after application (time between PRP application and questionnaire)
Improved group			
#1	19	16	16 (52 months)
#2	17	12	12 (55 months)
#3	20	11	11 (48 months)
#4	17	12	9 (55 months)
#5	17	14	17 (59 months)
#6	16	13	NA
#7	19	12	NA
Failed group			
#8	13	11	3 (62 months)
#9	19	17	17 (59 months)
#10	18	18	18 (52 months)
#11	19	19	19 (59 months)
#12	12	18	17 (57 months)
#13	14	18	18 (59 months)
#14	14	17	12 (53 months)
#15	8	17	17 (59 months)
#16	20	20	NA
#17	17	20	NA
#18	16	20	NA
#19	19	19	NA
#20	19	20	NA

NA: Six patients did not respond to the questionnaire since 2 (#6 and #16) could not be reached, 4 (#7, #18, #19 and #20) died, and 1 (#17) had a stoma

The primary focus of this study was to assess the efficacy of PRP injection in improving the Wexner score by a minimum of 2 points. We opted this scoring system and threshold level as previous studies have suggested that the Wexner score is the most suitable measure for assessing the severity of fecal incontinence, and 2 point improvement is deemed clinically significant [23, 24]. Although 35% of the study patients (*n* = 7) exhibited > 2 point improvement, comparison of pre- and post-injection Wexner scores did not reveal statistical significance. This may be because PRP did not work in some cases as several patients had the same or even worse Wexner scores after injection. Additionally, a smaller group of patients was able to discontinue the use of pads and antidiarrheal medications following PRP injection, while all patients had been using these items at the beginning of the study. The current information can be interpreted from two opposite perspectives. At first glance, one might conclude that PRP injection is not a viable option for incontinent patients due to the lack of significant difference between the pre- and post-injection Wexner scores. However, it is important to consider that a notable 35% of the cases did experience a clinically meaningful improvement in their incontinence scores, with some patients even reporting positive changes in their daily lives. Therefore, we believe that PRP injection has the potential to be helpful and warrants further investigation in future controlled trials.

Objective measures including manometry and EAUS were utilized for the evaluation of PRP injection to assess functional and anatomical changes, respectively. The manometric examinations revealed significant progress in squeezing pressure, indicating external sphincter function improvement following PRP injection. This is a promising outcome effect since it quantitatively demonstrates the functional effectiveness in such cases. These findings are in line with previous studies which have reported an improvement in parenchymal recovery and reduction in fibrotic scar size with PRP injection into the skeletal muscles [25–27]. However, our results failed to reveal statistical differences in pre- and post-injection EAUS measures for anatomical changes. This may be attributed to the challenge of identifying and measuring small changes in perianal muscle volumes. Additionally, manometric evaluation did not show a significant improvement in resting pressure. This could be due to the operation technique used on the patients as many underwent an intersphincteric resection that resulted in partial or total removal of internal sphincters. Additionally, even though our goal was to inject PRP directly into both internal and external muscles, some cases did not allow perfect visualization of the structures for injection due to narrower or defective internal muscles. However, we did observe that the improved group had slightly longer internal muscles, although the comparison was borderline insignificant (*p* = 0.057). Thus, we believe that an internal muscle reserve could be an important criterion for achieving better results after PRP injection. Furthermore, manometry examinations revealed a significant recovery in the first sensation with PRP injection. Although we are not sure of the exact reason for this finding, PRP may influence sensorial behaviors in the anal transitional zone. Further research is necessary to investigate this potential advantage of PRP in incontinent patients. Based on our research, use of PRP injections was found to positively impact certain aspects of QoL including physical functioning, role limitations, emotional well-being and social functioning. Nevertheless, it remains uncertain whether these improvements are attributable to the actual benefits of PRP treatment or simply a placebo effect given that patients are still taking medications and utilizing pads.

Long-term effect of PRP application was analyzed, and Wexner scores were evaluated at least 4 years after PRP application. Since four out of five patients who responded to the questionnaire reported stable Wexner scores, we believe that the improvement gained with PRP application remains secure in the long term in most of the patients who benefit at the beginning.

Current study aims to identify the factors that can predict the patients who would benefit from PRP application. The study found that type 2 diabetes was the sole significant aspect, as all three patients with type 2 diabetes reported a boost in their Wexner scores following PRP injection. Nevertheless, since there was only a limited number of patients with type 2 diabetes, the validity of this finding may be subject to debate.

A significant argument may be made regarding the clinical relevance of the improvement in Wexner scores after PRP injection. Although one-third of the patients declared > 2 points of progress in their Wexner scores, among this group, only one (5%) stopped using pads and three (15%) stopped taking antidiarrheal medications. So, we believe that although an improvement of > 2 points in Wexner scores in 35% of patients is a meaningful finding, the clinical consequence of PRP application in these cases may not be remarkable and has an impact on patients’ daily lives. This is probably a critical subject to be analyzed in further studies.

This study has certain strengths and limitations. The inclusion criteria were very selective, as PRP injection theoretically had the greatest impact on patients with weakened muscle strength. Therefore, patients with a loss of rectal compliance or insufficient neo-rectum volume identified during the manometer evaluation were excluded, while those with weakened perianal muscle strength were included as they were more likely to benefit from PRP injection. Another advantage is that the evaluation measures were multidimensional and included both patient-reported outcomes and several objective measures. Accordingly, the response to treatment was evaluated using QoL and incontinence questionnaires, as well as some objective measures including EAUS and anal manometer, to reveal anatomical and functional changes following PRP injection. The most significant limitation was the lack of a control group since the study was designed as a proof-of-concept trial. Besides, a study without a control group cannot rule out the potential effects of other causes on changes in QoL and improvement in continence over time after surviving cancer.

While the small sample size may be considered acceptable for an early phase or pilot study, it is also important to note that a few participants withdrew before undergoing manometry (*n* = 4) and EAUS (*n* = 3) 6 months after PRP application. Therefore, the final analyses for these measures were completed in 17 and 16 cases, respectively. Additionally, there is concern about confusing Wexner scores obtained at 6 months. Most patients remained stable or experienced limited or significant improvement in the severity of their incontinence; however, worse Wexner scores than initial ones were reported in some cases. A recent study on the expectations of community-living incontinent individuals regarding fecal incontinence treatments revealed that the highest priority of incontinent patients was complete elimination of fecal incontinence as well as a decrease in the amount and frequency of leaks [28]. Although the reason(s) for this unexpected outcome in the current study remains unclear, it might be related to higher expectations of the patients receiving PRP injection, and we cannot entirely dismiss the possibility of a true-negative impact of PRP on incontinence severity. Finally, the current study could not measure the effect of neoadjuvant radiation/chemoradiation on sphincter functions, since almost all patients received radiation before the operation. In addition, all patients were asked to continue regular pelvic exercises, and the benefit may be related to this practice.

In conclusion, considering the primary outcome measure, PRP did not improve the Wexner scores in fecal incontinence patients after rectal cancer surgery. However, administering PRP injection to perianal muscles may be advantageous for certain incontinent patients since this treatment can potentially improve their Wexner scores. The benefit in Wexner scores seems to continue in long-term follow-up. Additionally, some patients may no longer need to rely on medications or pads. PRP injection can improve the squeezing pressure and first sensation feeling in manometer examination as well as improve a few QoL measures. Nevertheless, further research is warranted to identify patient subpopulations that are most likely to benefit from PRP injection.

## Supplementary Information

Below is the link to the electronic supplementary material.

Supplementary file1 (DOCX 31 KB)

## Data Availability

No datasets were generated or analyzed during the current study.
